# Leadcare® II Comparison with Graphite Furnace Atomic Absorption Spectrophotometry for Blood Lead Measurement in Peruvian Highlands

**DOI:** 10.1007/s12291-022-01050-y

**Published:** 2022-05-30

**Authors:** Jaime Rosales-Rimache, Manuel Chavez-Ruiz, Jorge Inolopú-Cucche, Jhonatan Rabanal-Sanchez, Lenin Rueda-Torres, Gloria Sanchez-Holguin

**Affiliations:** 1 Instituto Nacional de Salud, Centro Nacional de Salud Ocupacional y Protección del Ambiente Para la Salud, Calle Las Amapolas 350, Lince, Lima, Perú; 2 Escuela Académico Profesional de Tecnología Médica, Universidad Privada Norbert Wiener, Lima, Perú

**Keywords:** LeadCare, Blood Lead, Graphite Furnace Atomic Absorption Spectrometry

## Abstract

Peru is one of the countries with the highest lead contamination in the world. Biological monitoring has limitations due to the shortage of laboratories with validated methodologies for the measurement of blood lead, and it is necessary to use alternative methods for its measurement in high-altitude cities. We aimed to compare the blood lead levels (BLL) measured by the LeadCare II (LC) method and Graphite Furnace Atomic Absorption Spectrometry (GF-AAS). We measured the BLL of 108 children from the city of La Oroya. The mean and median BLL for GF-AAS were 10.77 ± 4.18 and 10.44 µg/dL, respectively; for the LC method, the mean was 11.71 ± 4.28 and the median was 11.60 µg/dL. We found a positive linear correlation (Rho = 0.923) between both methods. Notwithstanding, the Wilcoxon test suggests a significant difference between both methods (*ρ* = 0.000). In addition, the Bland–Altman analysis indicates that there is a positive bias (0.94) in the LC method, and this method tends to overestimate the BLL. Likewise, we performed a generalized linear model to evaluate the influence of age and hemoglobin on BLL. We found that age and hemoglobin had a significant influence on BLL measured by the LC method. Finally, we used two non-parametric linear regression methods (Deming and Passing-Bablok regression) to compare the LC method with the GF-AAS. We found that these methods differ by at least a constant amount, and there would be a proportional difference between both. Although in general there is a positive linear correlation, the results of both methods differ significantly. Therefore, its use in cities located at high altitudes (higher than 2440 m.a.s.l.) would not be recommended.

## Introduction

Lead is a heavy metal with neurotoxic activity [1], and its chronic exposure could cause issues such as encephalopathy, peripheral neuropathy, hearing loss, or neurobehavioral deficits [2]. These conditions affect children more severely, generating an irreversible deficit in their cognitive development [3]. Environmental lead pollution is mainly linked to mining and metallurgical activities (foundries) that generate lead particles that contaminate water sources and farmland [4]. These operations occur in developing countries whose extractive activities are the basis of their economy [5].

Peru is one of the countries with the highest environmental lead contamination in the world, due to intense mining activity (3, 6). It has affected the inhabitants of Junín and Pasco for decades, located at more than 3000 m above sea level in the central Andes of Peru [6, 7]. Moreover, these regions do not have the infrastructure to implement gold standard methodologies for the measurement of blood lead levels (BLL), such as Graphite Furnace Atomic Absorption Spectrometry (GF-AAS) or Spectrometry Plasma Mass Analysis (ICP-MS) [3, 8]. It affects the monitoring and rapid response capacity of health authorities in the population exposed to lead. Because of this, the use of rapid BLL measurement devices was considered feasible [8, 9]. Among them, the LeadCare® II (LC) system is an instrument that allows fast and reliable measurement of BLL [10, 11]. It presents an optimal concordance with the results provided by GF-AAS [12–14]. However, the manufacturer does not recommend its use in cities localized at an altitude higher than 2440 m.a.s.I [15]. Given this, considering the limited evidence of the use of LC in field studies in high altitude conditions [16].

Our study aimed to compare the results of the LC (test method) with the GF-AAS (reference method) generated at more than 3500 m.a.s.l. Because of this, the use of devices for the rapid and reliable measurement of BLL in high-altitude cities is essential to improve primary and secondary prevention strategies in populations with risk lead exposure.

## Materials and Methods

### Population and Sample

108 children between 6 months and 12 years old with at least 6 months of residence in the city of La Oroya (Junín Region), whose approximate altitude is 3745 m above sea level, were enrolled. This city is exposed to lead due to intense mining activity [3]. The environmental temperature and relative humidity of this city was oscillating between 15 and 17 °C and 60–65%, respectively. Our study was carried out in 2016.

### Collection of Blood Samples

We obtained blood samples by finger puncture in heparinized capillaries and venipuncture in 3 mL vacuum tubes with EDTA K2. Finger blood samples were used for the determination of hemoglobin using the HemoCue® system and BLLs using LeadCare® II in situ. Venous blood samples were transferred in a cold chain (2–8 °C) to the toxicological laboratory of the National Institute of Health in Lima-Peru, which has international accreditation ISO / IEC 15,189 (EMA, certificate NMX-EC- 15,189-IMNC-2015), for determination of BLL by GF-AAS.

### Measurement of Blood Lead Levels (BLL)

As a test method, we used the portable LeadCare® II device, based on Anodic Stripping Voltammetry (ASV), which has a linearity range of 3.3 to 65 µg / dL (12). Before the analysis, we carried out self-calibration and internal quality control, following the manufacturer's specifications. The BLL measurement took 108 s and was performed immediately after obtaining the blood sample by fingerstick. On the other hand, as a reference method, we used Perkin Elmer equipment, model AAnalyst 800, based on the GF-AAS double beam methodology with Zeeman background correction and automatic sampler. We executed the method according to the MTA/MB-011 / R92 standard of the Spanish National Institute for Safety and Health at Work [17].

### Statistical Analysis

We performed a descriptive analysis of BLLs based on measures of central tendency and they were stratified according to age group and presence of anemia. The normality of the data distribution was evaluated using the Shapiro–Wilk test. The LC and GF-AAS methods were compared using Wilcoxon's non-parametric test and their correlation was determined using Spearman's Rho. The BLLs obtained by LC and GF-AAS were transformed on a logarithmic scale to fit a normal distribution. A generalized linear model (GLM) was used to evaluate the influence of age and hemoglobin concentration on the BLL of both methods and bias was calculated. A Bland–Altman graph was made using the logarithmic scale of the BLL as the regression line. Finally, Deming and Passing-Bablok regression analyses of BLL were performed on a logarithmic scale. For all statistical tests, a confidence level of 95% was considered. The data analysis was performed in the Stata Corp. Version 17 program and the plots were constructed using RStudio with ggplot function.

## Results

The BLL of both methodologies (LC and GF-AAS) present a similar mean, median, and standard deviation; however, the LC method has a higher interquartile range and a wide distribution of its values than the GF-AAS method (Table 1). Likewise, the data had a non-normal distribution (*ρ*-value < 0.05). Therefore, our study used non-parametric tests.

**Table 1:** Descriptive characteristics of the study variables (*n* = 108)

	Mean ± SD	CI (95%)	Median (IQR)	Min.–Max.	*ρ*-value^a^
Age (years)	4.96 ± 2.14	4.55–5.37	4 (3)	2–11	0.000
Hemoglobin (g/dl)	11.60 ± 3.76	10.89–12.33	10.67 (4.60)	7.04–26.05	0.000
BLL-LC (µg/dl)	11.71 ± 4.28	10.90–12.53	11.60 (5.65)	3.70–30.00	0.001
BLL-GF-AAS (µg/dl)	10.77 ± 4.18	9.97–11.57	10.44 (5.30)	3.04–26.05	0.001

^a^Shapiro-Wilk test. CI, Confidence interval. IQR, Interquartile Range. SD, Standard deviation

**Fig. 1:**
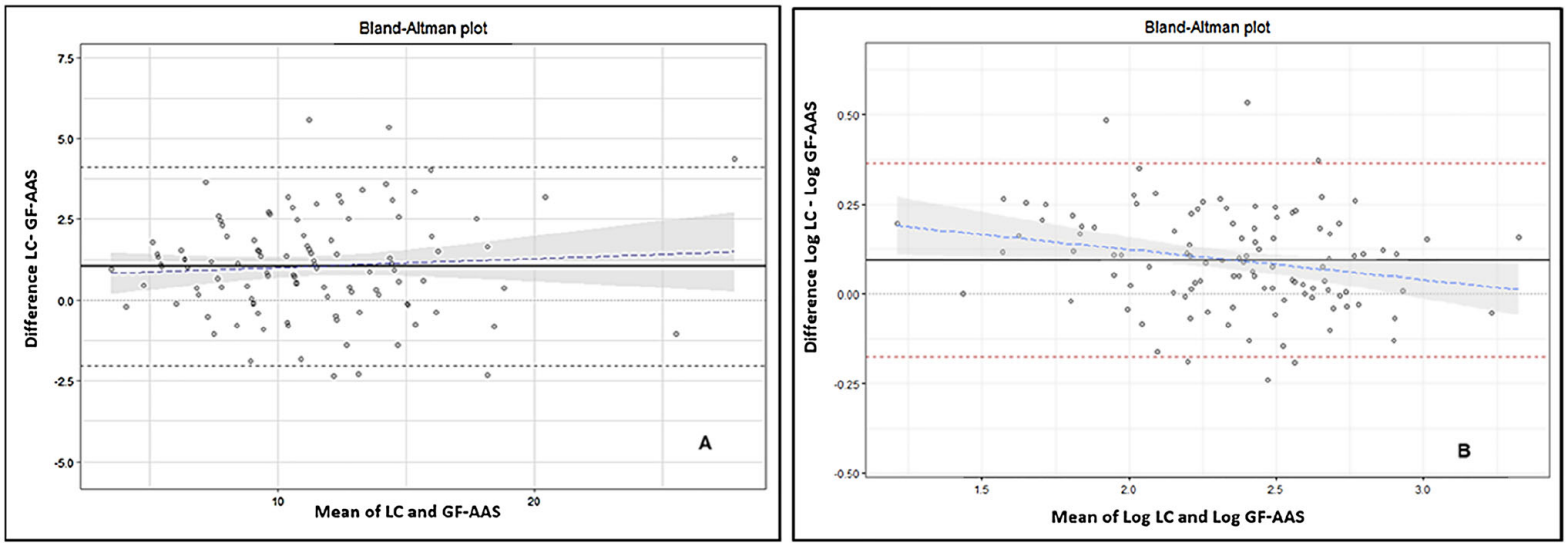
Bland–Altman plot between BLL-LC and BLL-GF-AAS (**A**). Bland–Altman plot between BLL-LC and BLL-GF-AAS on a logarithmic scale (**B**)

The Spearman correlation coefficient of BLL showed a positive linear correlation between the LC and GF-AAS methods (Table 2) and this correlation was statistically significant (*ρ*-value < 0.05). Furthermore, the bias between both methods was 0.940 (Table 2), which shows that the GF-AAS method measures, on average, 0.94 µg/dl less than the LC method. The Bland–Altman plot of BLL showed that 95% of the differences ranged from − 2.09 to 3.97 µg/dl. In contrast, on a logarithmic scale, it was − 0.18 and 0.36 µg/dl (Fig. 1).

**Table 2:** Correlation and bias between LC and GF-AAS (*n* = 108)

	Rho^a^	Bias(CI 95%)^b^	*p*-value^c^
BLL GF-AAS versus LC	0.923	0.940 (0.645–1.236)	0.000
BLL GF-AAS versus LC (< 6 years)	0.901	0.832 (0.428–1.237)	0.000
BLL GF-AAS versus LC (≥ 6 years)	0.753	1.156 (0.780–1.532)	0.000
BLL GF-AAS versus LC (with anemia)	0.920	1.070 (0.780–1.361)	0.000
BLL GF-AAS versus LC (without anemia)	0.930	0.744 (0.132–1.355)	0.034

^a^Spearman Correlation. ^b^Bland–Altman test. ^c^Wilcoxon test

The correlation was also estimated by age groups and anemia. We found that children aged 6 years and over presented a lower positive linear correlation than the group under 6 years of age; although its bias was greater (Table 2). On the other hand, a correlation was obtained in children with and without anemia, although in the latter group the bias was greater (Table 2). The Wilcoxon test of BLL showed that there are significant differences between the BLL obtained by the LC and GF-AAS methods (*ρ*-value < 0.05); Similar results were found in the categories according to age group and anemia (Table 2).

**Fig. 2:**
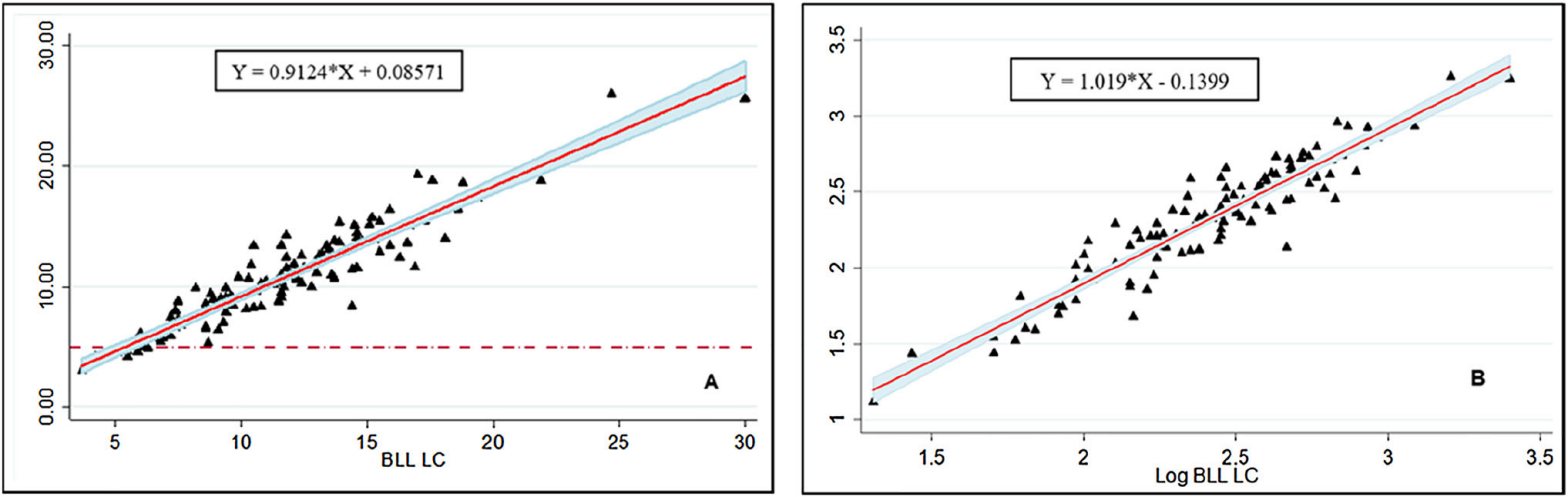
Scatter plot between BLL-LC and BLL-GF-AAS (**A**). Scatter plot between BLL-LC and BLL-GF-AAS on a logarithmic scale (**B**)

On the other hand, the dispersion of the data is close to the bisector and increases at lead concentrations above approximately 10 µg/dL (Fig. 2) and is consistent with the increase in the confidence interval. The BLL, on a logarithmic scale, presented an overestimation in lead concentration obtained by the GF-AAS method compared to the LC method. It can also be seen that the dispersion is reduced and remains close to the bisector, and the confidence intervals become narrower (Fig. 2).

Our study found that age and hemoglobin concentration were factors that significantly influenced (*ρ*-value < 0.05) in BLL obtained by the LC method. It was observed that, for each year of age, BLL increased by 0.201 µg / dL, while for each 1 g / dL of hemoglobin, BLL increased by 0.847 µg/dL (Table 3). Moreover, the hemoglobin concentration was a factor that significantly influenced (*ρ*-value < 0.05) in BLL obtained by the GF-AAS method; for every 1 g / dL of hemoglobin, BLL increased by 0.852 µg / dL.

**Table 3:** Age and hemoglobin influence on LC and GF-AAS methods

		Coefficient	CI 95%	*p*-value^a^
BLL LeadCare	Age (years)	0.201	0.009–0.393	0.040
	Hemoglobin (g/dl)	0.847	0.753–0.941	0.000
BLL GF-AAS	Age (years)	0.101	− 0.071–0.272	0.250
	Hemoglobin (g/dl)	0.852	0.768–0.936	0.000

^a^ GLM Gaussian family and link identity. CI- Confidence interval

The BLL were compared, on a logarithmic scale, obtained by the LC and GF-AAS methods, using two non-parametric tests (Deming and Passing-Bablok regression analyses). In both tests (Table 4) we found that the confidence interval of the intercept does not include unity and the slope does not include zero; therefore, both methods differ by at least a constant amount and there would be a proportional difference between both.

**Table 4:** LC and GF-AAS methods comparison

	Intercept	CI(95%)	Slope	CI(95%)
Passing-Bablok regression	− 0.307	− 0.487 to − 0.137	1.093	1.016–1.169
Deming regression	− 0.295	− 0.456 to − 0.135	1.084	1.018–1.150

CI- Confidence interval

**Fig. 3:**
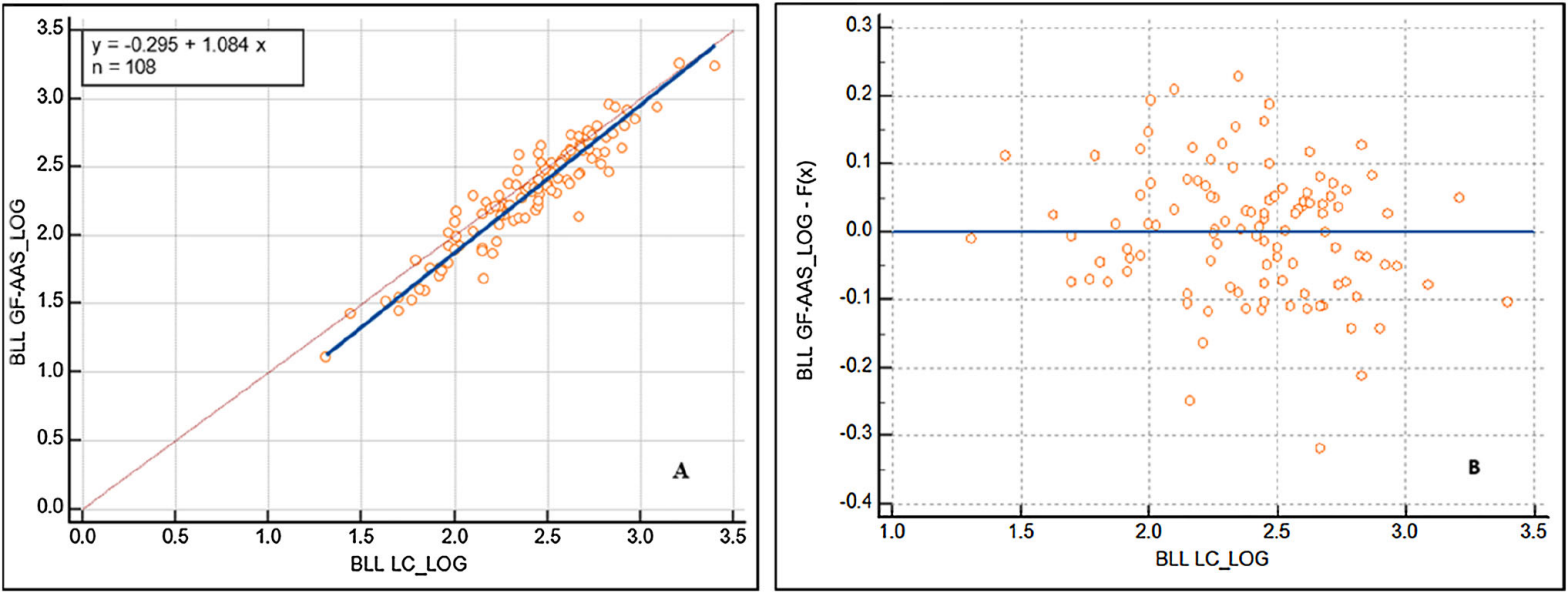
Deming regression between BLL-LC and BLL-GF-AAS on a logarithmic scale (**A**). Residual plot of the linear model (**B**)

**Fig. 4:**
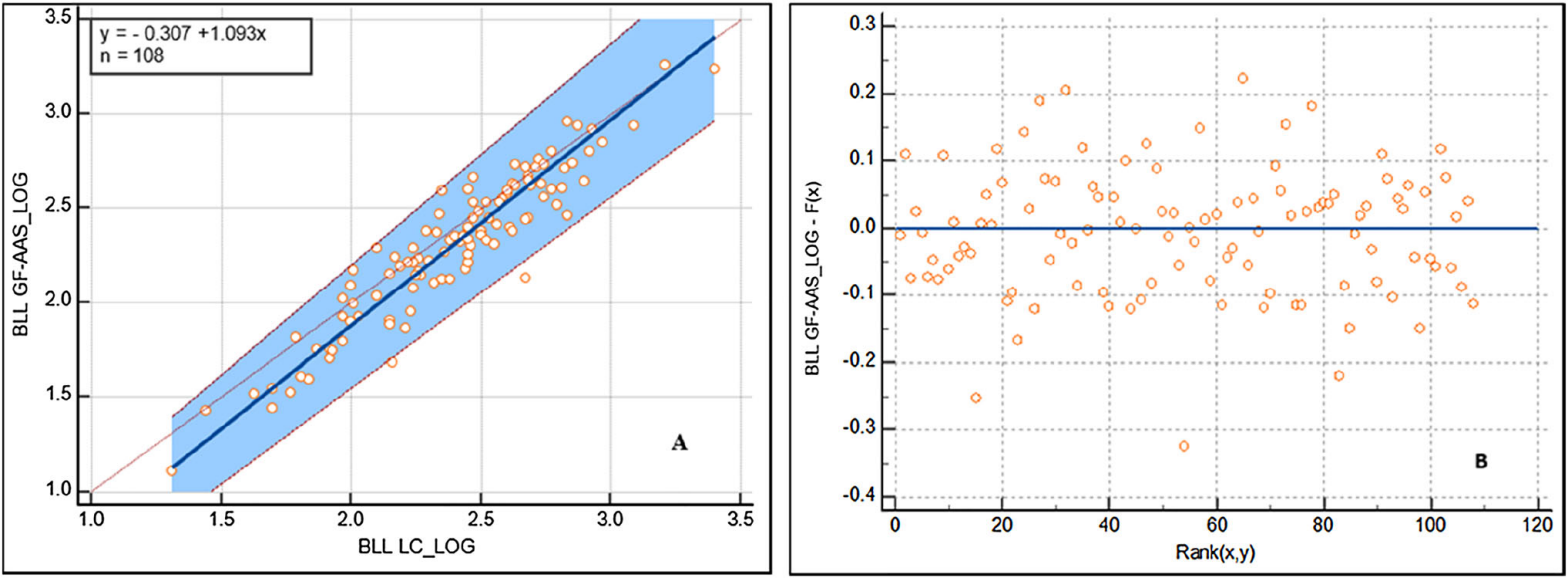
Passing-Bablok regression between BLL-LC and BLL-GF-AAS on a logarithmic scale (**A**). Residual plot of the linear model (**B**)

Finally, our study found a positive linear correlation between the LC and GF-AAS methods when they were evaluated by Deming and Passing-Bablok regression analyses (Figs. 3 and 4). In addition, the residuals did not present a determined pattern, so this would support the fact that there is a linear relationship between both methods.

## Discussion

Our study found a positive linear correlation between BLL measured with LC and GF-ASA, even between different age groups and the presence of anemia. However, the BLL obtained by the LC method was more dispersed than those of the GF-AAS method, which is related to what was observed in the Bland–Altman plot. A positive bias was found indicating that the LC method tends to overestimate BLL compared to the GF-AAS method. Our results are consistent with recent studies, which evaluated bias when comparing the LC method with the ICP-MS method, observing that this overestimation is evident above 10 µg/dL [10, 18].

On the other hand, our results indicate that the bias was greater in the group of children without anemia and in the group of children over 6 years of age. This is related to the results we found when evaluating the influence of age and hemoglobin on BLL measured with both methods. Using a generalized linear model, we found that age and hemoglobin levels influence the results obtained by the LC method. Hemoglobin levels also influence the results obtained with the GF-AAS method. This could be because chronic lead exposure is associated with anemia since lead interferes with heme biosynthesis [19].

Finally, the correlation analyzes using Deming and Passing-Bablok regression showed that the BLL in logarithmic scale obtained with both methods differ by at least a constant amount and there is at least a proportional difference between them. In this regard, one study found a linear correlation between the LC and ICP-MS methods, but the LC method presented a low bias in BLL close to the current reference level (5 µg/dl). Therefore, they recommended its use as a screening method for populations with low exposure to lead [10].

It has been reported that blood glutathione levels present in people living at high altitudes can interfere with BLL measurement when using the LC method. This is because the sulfhydryl groups of glutathione could bind to the surface of the electrode and block the active sites of equipment using ASV [16]. In this regard, a study in Peruvian workers working at 3800 m.a.s.l. reported that the measurements of the LC method are significantly different from those carried out by the GF-AAS [16].

Therefore, our results showed that the LC method did not provide significantly similar results to those obtained with GF-AAS in children living in the city of La Oroya, Junín region, Peru. This finding generates the need to guarantee the use of GF-AAS for the measurement of BLL, as well as the implementation of toxicology centers in the macro-regions of Peru. This should be in accordance with what is recommended by the World Health Organization (WHO), to improve strategies for primary and secondary care in the face of environmental exposure by lead [20], and in accordance with national policies that seek to strengthen the capacities of the Peruvian National Institute of Health [21]. Finally, we suggest that in subsequent studies a correction factor adjusted for altitude, age and sex be estimated, given that they are potential confounders identified. Its inclusion within an adjusted model could generate valid and reliable BLL by the LC method.

## Limitations

Among the most important limitations is the difference between the biological matrices used for the GFAAS and LeadCare methods, with venous and capillary blood, respectively; which could have generated variations in the results. Likewise, the venous blood samples had to be transported to the city of Lima for evaluation by GF-AAS, owing to the city of La Oroya does not have the infrastructure to perform this analysis.

## Conclusion

In conclusion, our study found that there is a positive linear correlation between BLL measured by the LC method and BLL measured by the GF-AAS method. Nonetheless, a positive bias and an overestimation of BLL measured by the LC method were found. Besides, the BLL of both methods show a significant difference and differ by at least a constant amount. Therefore, the use of the LC method would not be recommended in cities above 3500 m.a.s.l.
